# HybridTiger: Hybrid Model Checking and Domination-based Partitioning for Efficient Multi-Goal Test-Suite Generation (Competition Contribution)

**DOI:** 10.1007/978-3-030-45234-6_26

**Published:** 2020-03-13

**Authors:** Sebastian Ruland, Malte Lochau, Marie-Christine Jakobs

**Affiliations:** 8 University of Paderborn, Paderborn, Germany; 9 ICREA, Open University of Catalonia, Barcelona, Spain; 10 Technical University of Darmstadt, Department of Electrical Engineering and Information Technology, Real-Time Systems Lab, Darmstadt, Germany; 11 Technical University of Darmstadt, Department of Computer Science, Semantics and Verification of Parallel Systems, Darmstadt, Germany

**Keywords:** CPAchecker, Test-Goal Set Partitioning, Hybrid Model-Checking Cooperation

## Abstract

In theory, software model checkers are well-suited for automated test-case generation. The idea is to perform (non-)reachability queries for the test goals and extract test cases from resulting counterexamples. However, in case of realistic programs, even simple coverage criteria (e.g., branch coverage) force model checkers to deal with several hundreds or even thousands of test goals. Processing each of these test goals in isolation with model checking techniques does not scale. Therefore, our tool HybridTiger builds on recent ideas on multi-property verification. However, since every additional property (i.e., test goal) reduces the model checker’s abstraction possibilities, we split the set of all test goals into different partitions. In Test-Comp 2019, we applied a random partitioning strategy and used predicate analysis as model checking technique. In Test-Comp 2020, we improved our technique in two ways. First, we exploit domination information among control-flow locations in our partitioning strategy to group test goals being located on (preferably) similar paths. Second, we account to inherent weaknesses of the predicate analysis by applying a hybrid software model-checking approach that switches between explicit model checking and predicate-based model checking on-the-fly. Our tool HybridTiger is integrated into the software analysis framework CPAchecker.

## 1 Software Architecture

The HybridTiger algorithm is implemented within the software verification framework CPAchecker [4]. CPAchecker utilizes the Eclipse CDT C-parser[3].

[3] https://www.eclipse.org/cdt/

```c
int fib(int n){
  if(n <= 0) return -1;
  if(n == 1) return 1;
  if(n == 2) return 1;
  return fib(n-1)
        +fib(n-2);
}
```

(a) C-Program

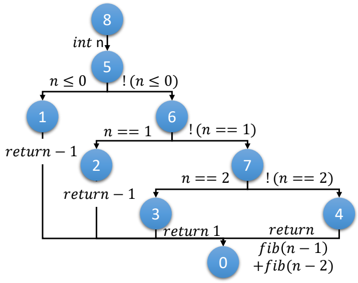

(b) CFA

**Fig. 1.** C Program to calculate the Fibonacci number of $n$ and corresponding CFA

CPAchecker allows developers to easily integrate new algorithms like HybridTiger, which may use other algorithms implemented in CPAchecker, such as counterexample-guided abstraction refinement (CEGAR) [5]. Additionally, new reachability analyses can be integrated as Configurable Program Analyses (CPAs) [2]. Each CPA consist of an abstract domain with the operators *post*, *merge*, and *stop*. Multiple CPAs can also be combined into one CPA.

HybridTiger uses the CoVeriTest [3] algorithm to sequentially combine test-case generation runs utilizing different verification techniques. Each test-case generation run applies the CPA/Tiger-MGP[4](Tiger Multi-Goal-Partitioning) algorithm, which utilizes the CEGAR algorithm.

## 2 Test-Generation Approach

HybridTiger first extracts test goals from input programs and repeatedly executes reachability analyses provided by CPAchecker until every reachable test goal is covered by at least one test case. To this end, test goals are encoded into (non-)reachability properties. If a test goal has been reached, CPAchecker thus returns a counterexample and HybridTiger extracts a test case (i.e., a vector of input values), writes the test case to disk and marks the test goal as covered.

*Hybrid Test-Case Generation.* HybridTiger receives as inputs a C program and a property specification (i.e., a set of test goals). Next, HybridTiger transforms the C program into a control-flow automaton (CFA) [1]. Figure 1 shows an example C program and the corresponding CFA. After CFA generation, the CoVeriTest algorithm as configured in HybridTiger (see Fig. 2) is executed. In every new iteration, each analysis of our configuration first (re-)partitions the set of uncovered test goals (e.g., partitions P1, P2, P3 and P4 for CPA/-Tiger-MGP-Value and P1 and P2 for CPA/Tiger-MGP-Predicate in Fig. 2). In each iteration, CPA/Tiger-MGP-Value is performed first using explicit model checking and is stopped after 120s. After that, CPA/Tiger-MGP-Predicate is

[4] https://www.es.tu-darmstadt.de/es/team/sebastian-ruland/testcomp19/

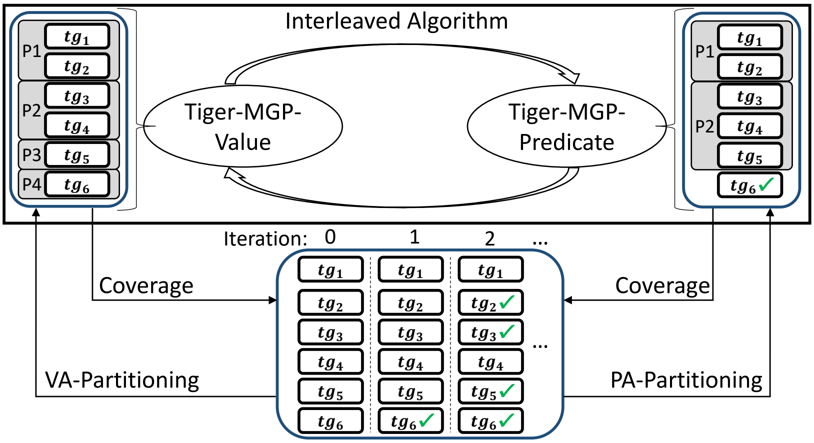

**Fig. 2.** Overview of HybridTiger executed using predicate model checking for 780s, where the overall iteration stops after reaching the global time limit.

*Partitioning.* HybridTiger utilizes domination information of test-goal locations according to the respective CFA paths. This meta-information is retrieved from the generated CFA: each CFA node (i.e., basic block of program locations) in Fig. 1 is annotated with a *post-order ID* such that a node will only be reached *after* all nodes on the same path with a larger ID have been reached at least once. Hence, we use the IDs of predecessor nodes related to the CFA edges of test goals as sorting criterion for the overall set of test goals before splitting this set into partitions of predefined sizes. In this way, test goals sharing similar paths are more likely to be assigned to the same partition thus facilitating reuse potentials of reachability-information during reachability analysis.

## 3 Strengths and Weaknesses

HybridTiger has three main strengths. First, the *directed* generation of test cases aiming at covering particular test goals significantly reduces the overall number of test cases. Additionally, most test cases produced by HybridTiger effectively increase the overall coverage (i.e., HybridTiger produces mostly correct and non-redundant test cases). Second, HybridTiger uses control-flow information to partition test goals which potentially enhances efficiency of test-case generation due to information reuse among similar test goals. Lastly, HybridTiger uses combinations of different analysis strategies (i.e., value analysis and predicate analysis) to cope with structural diversity of input programs. One weakness of HybridTiger is that the partitioning approach does not improve performance of a goal-by-goal approach if being applied to programs with a small number of test goals (e.g., reaching one single error location as demanded in the Cover-Error category).

*Results.* In Test-Comp 2020, HybridTiger has participated in all categories and managed to reach the 4th rank in *Code Coverage* and the 6th rank in *Finding Bugs*, where HybridTiger performed better on tasks with many test goals.

## 4 Setup and Configuration

The version of HybridTiger submitted to Test-Comp 2020 is built from the tigerIntegration2[5] branch revision 32283 of the CPAchecker repository and is archived at https://gitlab.com/sosy-lab/test-comp/archives-2020. HybridTiger can be applied to a single file using the command

```
scripts/cpa.sh -benchmark -heap 10000M -tigertestcomp20
    -spec spec.prp file
```

where *spec* is the property file (e.g., *coverage-error-call* or *coverage-branches*) and *file* is the input C program. Statistics of the analyses are printed to console and meta data on generated test cases as well as the test suite are written to files in the output folder. In order to run HybridTiger for the Test-Comp 2020 benchmarks a Linux system with Java 8, BenchExec[6] and the SV-benchmarks[7] is required. Finally, run *BenchExec* with:

- the benchmark definition *cpa-tiger.xml* (archived at https://gitlab.com/sosy-lab/test-comp/bench-defs/tree/master/benchmark-defs), and
- the tool-info module *cpachecker.py* (archived at https://github.com/sosy-lab/benchexec/tree/master/benchexec/tools).

## 5 Project and Contributors

CPAchecker is maintained by the Software Systems Lab at LMU Munich as open-source project, contributed by an international group of researchers from LMU Munich, University of Passau, Technical University of Darmstadt and the Institute for System Programming of the Russian Academy of Sciences.The branch *tigerIntegration2* from which HybridTiger is built is mainly developed at the Technical University of Darmstadt. Additional information is available at https://cpachecker.sosy-lab.org/.

*Acknowledgement.* This work was funded by the Hessian LOEWE initiative within the Software-Factory 4.0 project.

5 https://svn.sosy-lab.org/software/cpachecker/branches/tigerIntegration2
6 https://github.com/sosy-lab/benchexec
7 https://github.com/sosy-lab/sv-benchmarks

## References

[1] Beyer, D., Cimatti, A., Griggio, A., Keremoglu, M., Sebastiani, R.: Software model checking via large-block encoding. In: 2009 Formal Methods in Computer-Aided Design. pp. 25–32 (12 2009)

[2] Beyer, D., Henzinger, T.A., Théoduloz, G.: Configurable Software Verification: Concretizing the Convergence of Model Checking and Program Analysis. In: Proc. CAV, LNCS 4590. pp. 504–518. Springer Berlin Heidelberg (2007)

[3] Beyer, D., Jakobs, M.C.: CoVeriTest: Cooperative Verifier-Based Testing. In: Proc. FASE. pp. 389–408. Springer International Publishing (2019)

[4] Beyer, D., Keremoglu, M.E.: CPAchecker: A Tool for Configurable Software Verification. In: Proc. CAV, LNCS 6806. pp. 184–190. Springer Berlin Heidelberg (2011)

[5] Clarke, E., Grumberg, O., Jha, S., Lu, Y., Veith, H.: Counterexample-guided Abstraction Refinement for Symbolic Model Checking. J. ACM **50**(5),752–794 (2003)

